# Supplementary material for: Evaluating the Assembly Strategy of a Fungal Genome from Metagenomic Data: Solorina crocea (Peltigerales, Ascomycota) as a Case Study
Source: J Fungi (Basel). 2025 Aug 15;11(8):596. doi: 10.3390/jof11080596 (PMC12387558; doi:10.3390/jof11080596)
Supplement: Supplementary file 1 [file jof-11-00596-s001.zip › jof-3760483-supplementary materials.pdf]

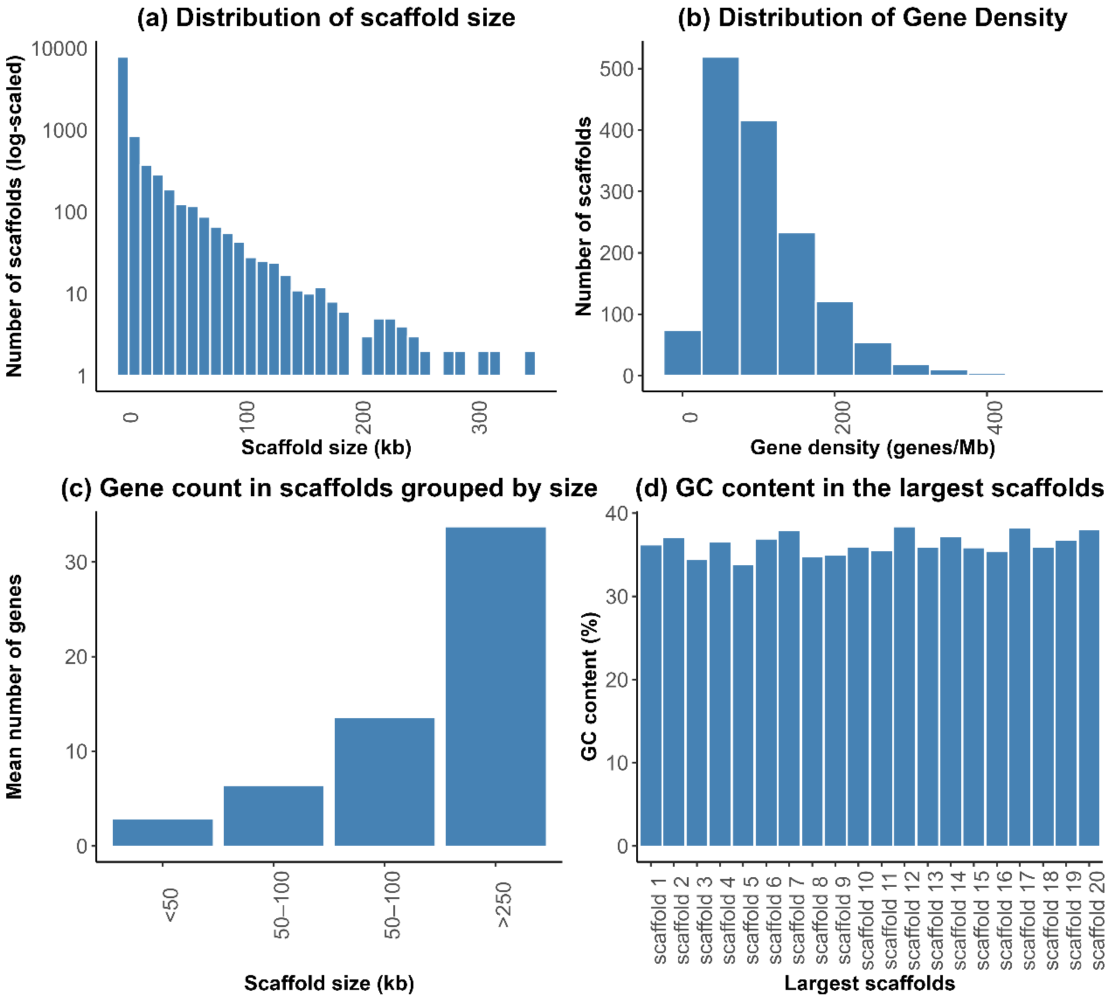

Figure S1. Characteristics of the genome assembly obtained with the Strategy 1. (a) Distribution of scaffold sizes and (b) distribution of gene density, (c) number of genes found in scaffolds grouped by size and (d) GC content of the largest scaffolds. The size of the largest scaffolds shown in the figure ranged from 209.3 to 347.6 kb.

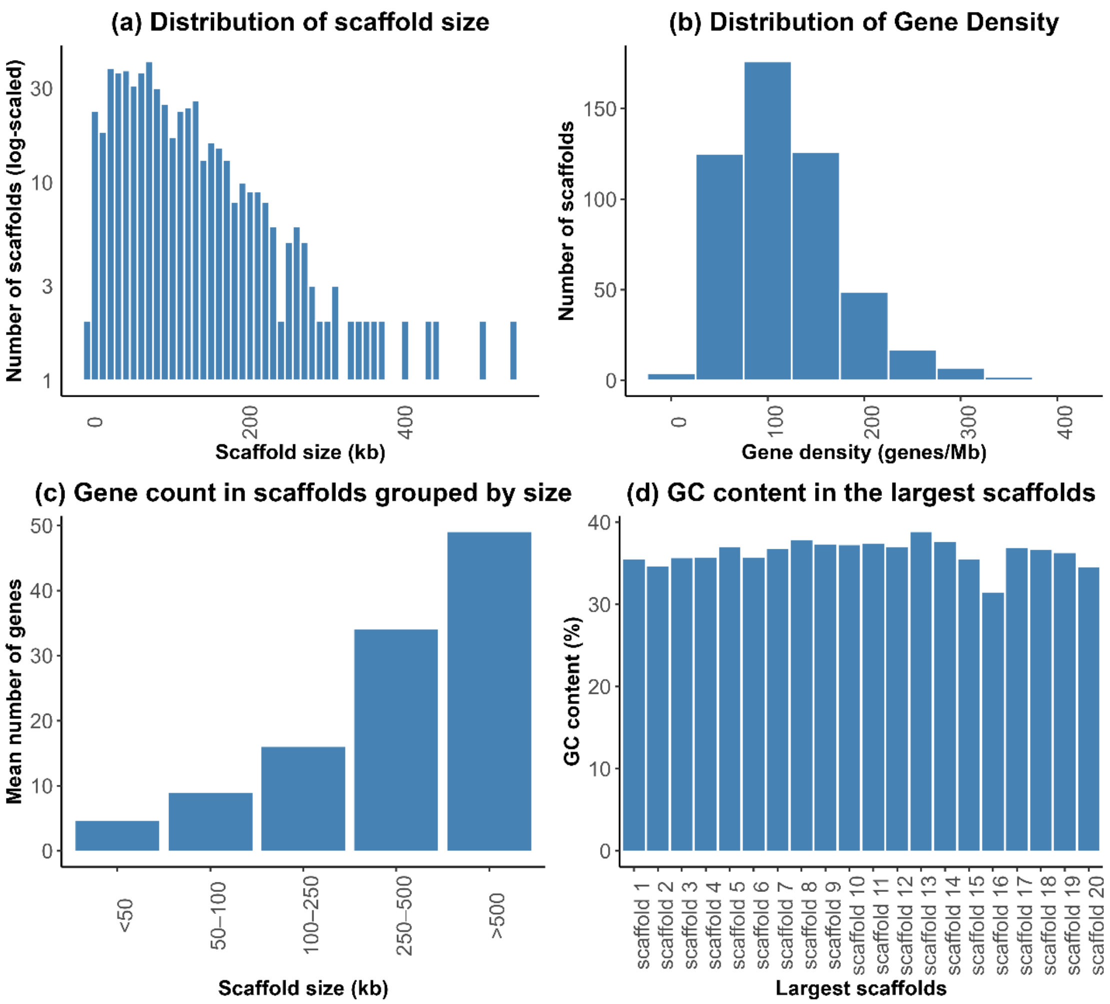

Figure S2. Characteristics of the genome assembly obtained with Strategy 2. (a) Distribution of scaffold sizes and (b) distribution of gene density, (c) number of genes found in scaffolds grouped by size and (d) GC content of the largest scaffolds. The size of the largest scaffolds shown in the figure ranged from 276.1 to 547.6 kb.

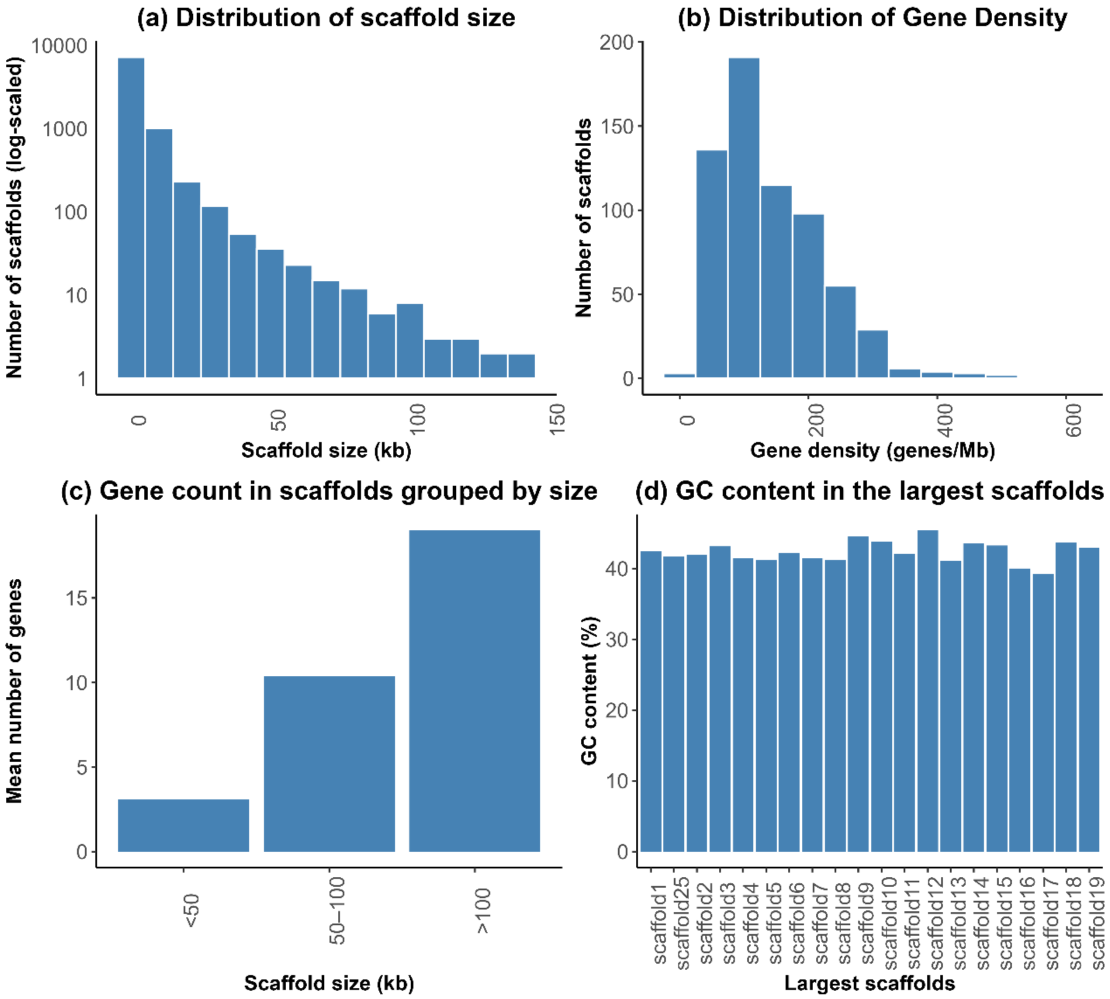

Figure S3. Characteristics of the genome assembly obtained with Strategy 3. (a) Distribution of scaffold sizes and (b) distribution of gene density, (c) number of genes found in scaffolds grouped by size and (d) GC content of the largest scaffolds. The size of the 20 largest scaffolds shown in the figure ranged from 94.1 to 140.1 kb.

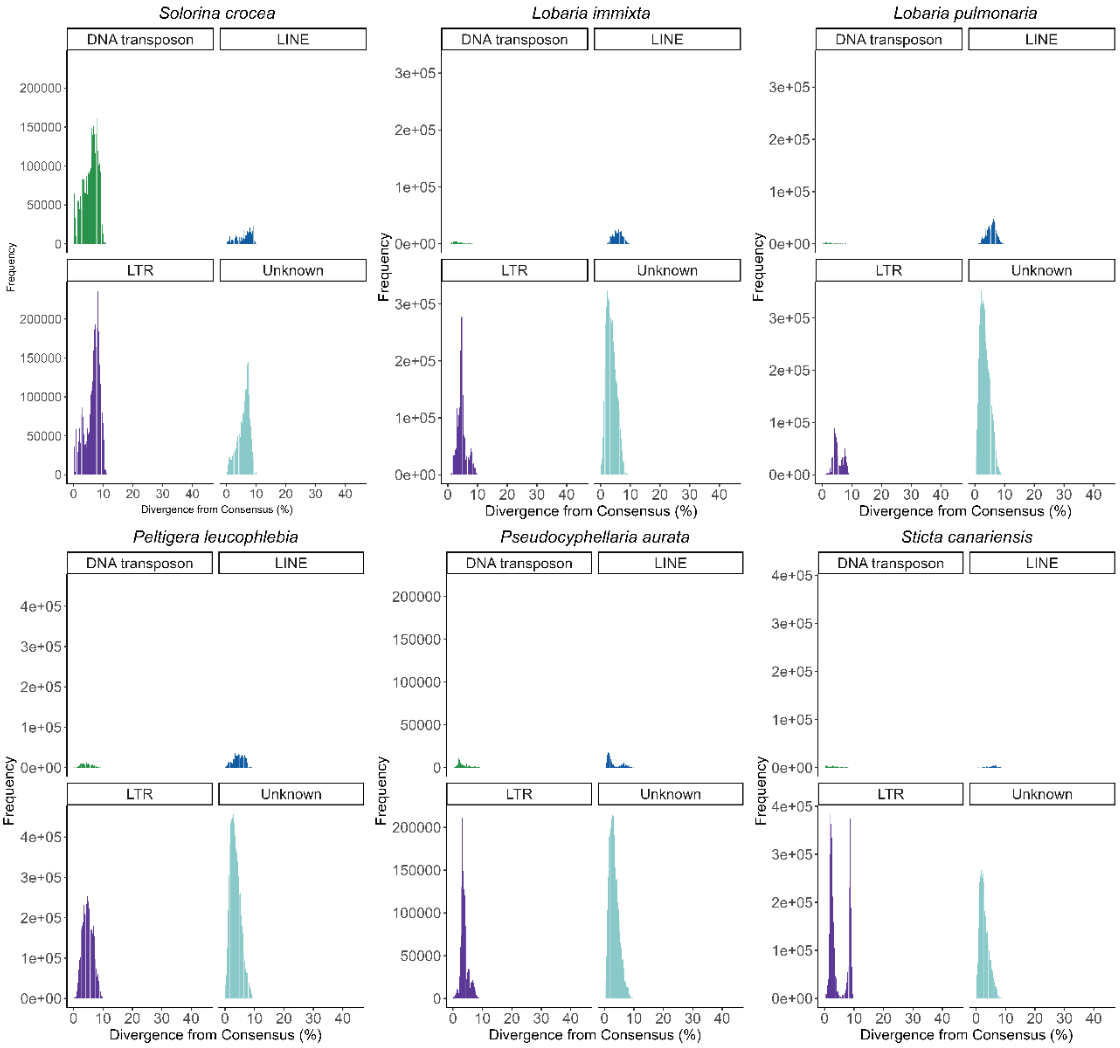

Figure S4. Repeat landscapes of the genomes of *S. crocea* and other available Peltigerales. Repeat landscapes of Peltigerales genomes gen5erated from the library of repetitive elements and their consensus sequences, for DNA transposons, LINEs, LTRs and unidentified repetitive elements.

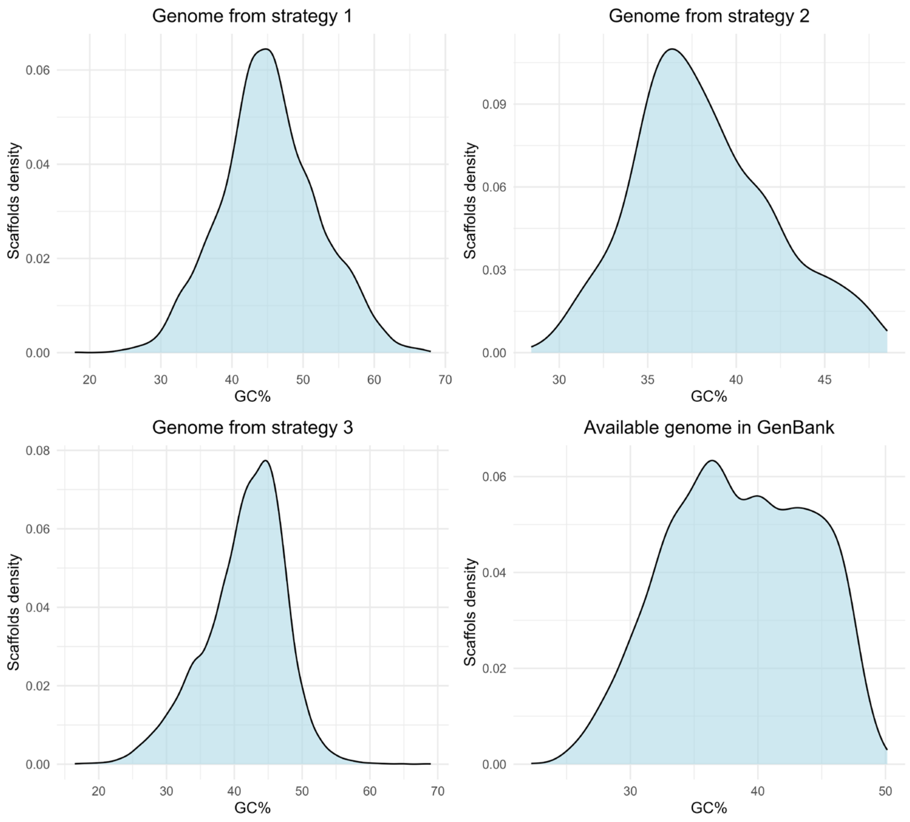

Figure S5. Distribution of GC content in the genomes obtained with Strategies 1, 2 and 3 and another genome of *Solorina crocea* previously published. It corresponds to the BioProject PRJEB77567.

Table S1. Statistics of available Peltigerales genomes. Metrics with asterisks indicates values at contig level. Accessions with asterisks indicates assemblies marked as reference genomes according to the database. All of them are metagenome-assembled genomes sequenced from thallus using Illumina technology.

Accession	Species	Size (Mb)	Scaffold N50 (kb)	No. Scaffolds	GC percent	No. Genes
GCA_964256785.1	*Coccocarpia palmicola*	20.54	11.91	2,068	47	-
GCA_964256385.1	*Collema furfuraceum*	21.05	6.10	3,967	48.5	-
GCA_964255535.1	*Dendriscocaulon intricatulum*	32.10	6.16	6,043	44.5	-
GCA_964256325.1	*Fuscopannaria leucosticte*	27.20	12.71	2,652	46.5	-
GCA_964255575.1	*Leptogium austromericanum*	29.58	63.37	720	47.5	-
GCA_964255685.1	*Leptogium austromericanum*	27.20	12.02	2,884	47	-
GCA_964254935.1	*Leptogium chloromelum*	22.42	8.96	3,001	47	-
GCA_964256315.1	*Leptogium corticola*	24.42	6.95	4,109	47	-
GCA_022814215.1*	*Lobaria immixta*	55.89	79.75	1,045	47	12,122
GCA_964257085.1	*Lobaria pulmonaria*	46.62	25.65	2,527	48	-
1006354	*Lobaria pulmonaria Scotland reference genome v1.0*	56.12	54.16	1,911	47.16	15,607
1166970	*Lobaria pulmonaria Africa extracted metagenome v1.0*	55.03	57.26	1,556	47.19	16,100
1166959	*Lobaria pulmonaria Scotland extracted metagenome v1.0*	55.85	81.69	1,189	47.15	16,471
1166966	*Lobaria pulmonaria Spain extracted metagenome v1.0*	55.60	75.12	1,257	47.20	16,296
1166971	*Lobaria pulmonaria Switzerland extracted metagenome v1.0*	54.93	67.84	1,405	47.21	16,426
GCA_964254995.1	*Nephroma helveticum*	28.73	11.62	3,120	47	-
GCA_964257215.1	*Pannaria tavaresii*	16.85	4.15	4,346	45	-
GCA_964257115.1	*Peltigera aphthosa*	88.82	23.39	5,641	38	-
GCA_964254465.1*	*Pectenia cyanoloma*	25.44	29.60	1,168	45.5	

GCA_964257155.1	*Peltigera dolichorrhiza*	73.93	34.01	3,393	35.5	-
GCA_964256605.1	*Peltigera evansiana*	98.57	95.17	2,350	35.5	-
GCA_964255975.1	*Peltigera extenuate*	72.13	44.80	2,838	37	-
GCA_964256015.1	*Peltigera hydrothyria*	130.5	46.55	4,665	24.5	-
GCA_022814155.1*	*Peltigera leucophlebia*	44.69	10.62	4,464	45.5	8,262
GCA_964255295.1*	*Pectenia plumbea*	26.11	43.31	882	45	-
GCA_964256585.1	*Peltigera phyllidiosa*	82.78	24.56	4,792	32.5	-
GCA_964256885.1	*Placynthium petersii*	21.22	10.38	2,580	47.5	-
GCA_022814125.1*	*Pseudocyphellaria aurata*	42.93	74.99	978	49	10,009
GCA_964257125.1	*Solorina crocea*	93.72	50.47	3,351	37	-
GCA_022814045.1*	*Sticta canariensis*	43.44	16.1*	3.190*	47	8,568
GCA_964254715.1	*Sticta sp.*	30.30	13.76	2,794	48.5	-

Table S2. Benchmarking of the content of repetitive elements in genomes resulting from Strategies 1, 2 and 3. Values represent the percentage of the genome that is covered by each group of repetitive elements.

Repetitive element	Strategy 1	Strategy 2	Strategy 3
Total retrotransposons	11.34	7.65	15.50
Retrotransposons:LINEs	2.84	0.64	3.57
Retrotransposons: LTR	8.50	7.01	11.93
LTR: Ty1/Copia	2.80	1.10	4.27
LTR: Gypsy/DIRS1	0.56	5.11	2.25
Total DNA transposons	10.26	5.85	5.82
Rolling-circles	0	0.68	0
Small RNA	0	0	0
Simple Repeats	0.59	0.62	0.53
Low Complexity	0.17	0.18	0.10
Unclassified	0.17	7.20	0.09

Table S3. Functional annotation of the genome of *Solorina crocea* obtained with the different strategies. Number of annotated terms received from each queried database after running the FunAnnotate pipeline.

Annotation Category	Strategy 1	Strategy 2	Strategy 3
BUSCO (Dikarya_odb9)	1,163	1,146	436
dbCAN	176	152	68

EggNog	6,367	11,866	4922
Pfam	7,835	7,996	3360
UniProtKB	681	4,644	885
MEROPS	195	178	68
Interpro	7,290	16,542	2705
GO terms	6,697	10,161	2167
Total annotated terms	30,404	52,648	14,611

Table S4. Abundance of repetitive elements in available Peltigerales genomes. Abundance of transposable elements of class I (retroelements) and class II (helitrons and other DNA transposons) represented with the percentage of the genome that is covered by them in each species. The asterisk indicates the genome sequenced in this study.

Species	% Covered genome			
	Retroelements	DNA transposons	Helitrons	Unclassified
*Solorina crocea**	7.65	5.85	0.68	7.20
Peltigera leucophlebia	11.83	0.48	0.30	18.12
Lobaria pulmonaria	2.88	0.05	0.03	10.13
Lobaria immixta	4.93	0.11	0.01	9.81
Pseudocyphellaria aurata	3.52	0.20	0	7.77
Sticta canariensis	8.11	0.13	0	8.59
